# Cross-linking mass spectrometry guided structure prediction of disordered *Encephalitozoon hellem* proteins

**DOI:** 10.1128/mbio.02261-26

**Published:** 2026-10-20

**Authors:** Elizabeth Weyer, Yongliang Wang, Tadakimi Tomita, Carlos Madrid-Aliste, Prithviraj Nandigrami, Andras Fiser, Simone Sidoli, Jennifer T. Aguilan, Bing Han, Louis M. Weiss

**Affiliations:** 1 Department of Pathology, Albert Einstein College of Medicine 2006, New York, New York, USA; 2 Department of Pathogenic Biology, School of Basic Medical Sciences, Cheeloo College of Medicine, Shandong University 600286, Jinan, Shandong, China; 3 Department of Biochemistry, Albert Einstein College of Medicine 548274, New York, New York, USA; 4 Department of Systems & Computational Biology, Albert Einstein College of Medicine 2006, New York, New York, USA; 5 Department of Genetics, Albert Einstein College of Medicine 2006, New York, New York, USA; 6 Department of Medicine, Albert Einstein College of Medicine 200531, New York, New York, USA; Georgia Institute of Technology, Atlanta, Georgia, USA

**Keywords:** microsporidia, protein structure, proteomics, cross-link mass spectrometry, protein complex, spore wall, host cell invasion

## Abstract

Microsporidia such as *Encephalitozoon hellem* are obligate intracellular human parasites that remain genetically intractable, limiting the functional characterization of their proteomes. Experimental structural studies are challenging, as many proteins in microsporidia comprise a large percentage of disordered regions. Computational structural studies based on homology-based modeling and the use of deep-learning algorithms for microsporidian proteins also remain limited because most have little to no sequence similarity to proteins with solved structures. To address these limitations, we developed an approach that incorporates cross-linking mass spectrometry (XL-MS) data into structure prediction. XL-MS data provide upper-bound distance constraints that can be incorporated into protein deep-learning-based modeling and subsequent docking. Using this approach, we generated a model of two interacting *E. hellem* spore wall proteins, spore wall protein 1B (Swp1b) and endospore protein 1 (EnP1), which have no clear homologs outside of microsporidia and contain several disordered regions. These proteins are abundant spore wall proteins of microsporidia and previously were not known to interact with one another. The resulting model not only is consistent with the experimental cross-links used to generate the model but was also subsequently confirmed by independently generated XL-MS data. The described AlphaLink-Modeller framework for structure prediction is particularly well suited to proteins with limited homology and/or substantial flexible regions, given that they adopt a defined structural state within a biological context, thereby extending integrative modeling approaches to previously inaccessible targets.

**IMPORTANCE:**

In this article, we demonstrate an improved method for the prediction of the structure of protein complexes with no clear homologs and a considerable number of disordered regions. This is significant because these types of proteins are critical to understanding the biology of organisms, yet model generation of these classes has been inaccessible. The method incorporates experimental data obtained from cross-linking mass spectrometry (XL-MS) into the modeling process, providing a method to integrate such experimental data into computational modeling of the structure of proteins and protein interactions. For this paper, we have used this method for predicting the structure of two highly abundant *Encephalitozoon hellem* proteins in the spore wall that are important for host cell infection due to this pathogen of humans and other vertebrates.

## INTRODUCTION

Microsporidia are a large family of intracellular pathogens that evolutionarily are related to the Cryptomycota and are probably a sister taxon to the fungi (1). They are highly divergent and have undergone extensive genome reduction and rapid sequence evolution, resulting in proteins that are structurally and functionally distinct and frequently lack recognizable homologs in other organisms and for which few structures have been solved (2, 3). Microsporidia are also genetically intractable, which limits the ability to use genetic modifications of genes to understand protein structure and function. In addition, many proteins in microsporidian invasion organelles of interest comprise a large percentage of disordered regions. As a result, the structure of many microsporidian proteins is difficult to predict, and it is also likely that X-ray crystallography would not be able to resolve the structures of these flexible regions.

Classical methods of protein modeling like homology modeling have struggled to predict structures of low-homology proteins, and *ab initio* modeling is limited by size. The AI boom has revolutionized protein modeling with the development of deep-learning-based models such as AlphaFold (4), RoseTTAFold (5), DeepFold (6), OmegaFold (7), and ESMFold (8). Millions of predicted protein structures are now available as models through AlphaFold DB (9). However, it is notable that many of these predicted structures contain domains or whole proteins that are of low confidence. Out of the 49 model organisms that have their entire proteome predicted, approximately 30–40% of all residues have low or very low confidence scores (pLDDT < 0.7) (10). This is in part because AlphaFold and other similar algorithms were trained on proteins with experimentally solved structures and large multiple sequence alignments (4). Proteins with few to no homologs provide weak multiple sequence alignments, so predictions of these proteins are not as reliable (11–14). Even single-sequence prediction algorithms like ESMFold that do not use multiple sequence alignments are less reliable for proteins with limited evolutionary signal and/or proteins with considerable flexible regions (11, 15, 16). In general, flexible regions also do not usually yield defined structures when examined with experimental methods such as X-ray crystallography and cryo-EM (17, 18).

More recently, efforts have been made to advance integrated structural modeling of proteins, where experimental data are combined with computational methods to determine the structures of proteins and their complexes (19–22). Cross-linking mass spectrometry (XL-MS) is a particularly useful tool for providing structural information for protein complexes with low homology and disordered regions. XL-MS uses MS-cleavable bifunctional cross-linking molecules that link two amino acids within a specific distance, thereby giving an experimental distance restraint on amino acid proximity (23). These cross-linking molecules are added in the native protein environment, and so they capture these distance restraints in an unbiased fashion. Links formed between proteins (interlinks) in close proximity can provide structural information on how two proteins interact. Links formed within individual proteins (intralinks) provide experimental distance restraints for the structures of the individual proteins in their native environments. These interlinks and intralinks are especially useful to inform modeling for proteins whose structures cannot be solved by traditional methods and for structures that struggle to be solved purely computationally. It has been integrated into existing tools like AlphaFold, docking pipelines, and atomistic simulations, making it generalizable for all proteins (24–31).

Employing XL-MS to examine the proteome of *E. hellem*, we discovered that the proteins spore wall protein 1B (Swp1b) and endospore protein 1 (EnP1) are in close proximity to one another, as evidenced by the finding of numerous interlinks between these proteins in XL-MS experiments. Microsporidia have a thick spore wall that is made up of three layers—an inner plasma membrane, a middle layer of protein (endospore), and an outside layer of protein (exospore) (32). EnP1 is a very abundant protein in the endospore. It is important to the structure of the wall, but when secreted into the host cell, it has been found to also have a role in modifying infected host cell gene expression (33, 34). Swp1b is a very abundant protein in the exospore and is involved in the adhesion of spores to host cells and in providing spore wall stability (35). These proteins could interact with each other at the interface of the endospore and exospore layers in the spore wall.

Due to their high abundance and importance in the spore wall structure, we were interested in modeling the interaction of Swp1b and EnP1, but these proteins are both very unique when compared to proteins in all other species and are predicted to have a high percentage of flexible regions. Due to their predicted flexible regions and large size, the structure of these proteins is expected to be difficult to solve by X-ray crystallography, cryo-EM, or NMR. In fact, crystal production of heterologously expressed structural microsporidian proteins has, in general, not been successful. When Swp1b and EnP1 proteins are modeled using AlphaFold, the models are inconsistent with our experimental distance restraints (i.e., the cross-links identified by XL-MS both between these proteins and the internal cross-links within each protein). Even introducing distance restraints into an AI pipeline (AlphaLink) results in structures still incompatible with the experimental data obtained by XL-MS (i.e., all of the cross-linked peptides identified). Thus, we developed an approach that combines XL-MS-guided AI modeling with XL-MS-guided refinement and docking to provide structures that are consistent with the observed experimental distance constraints. In this paper, we present a novel method that allows one to model flexible, non-homologous protein complexes previously unable to be resolved with purely experimental or computational methods.

## RESULTS

### Incorporating XL-MS data into modeling protein complexes

**Fig 1:**
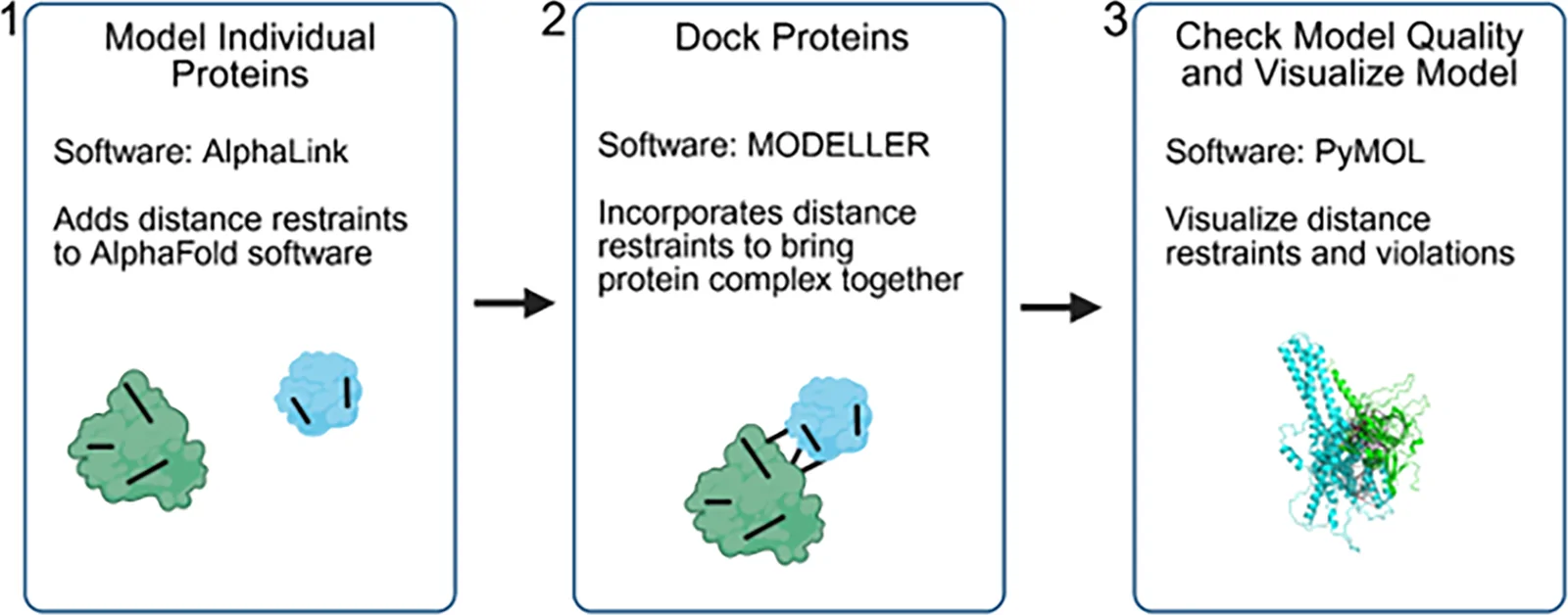
A new analytical approach for incorporating XL-MS data into modeling protein complexes. Each step of this three-step approach incorporates XL-MS data into its prediction, resulting in structures that are compatible with experimental XL-MS data, even proteins that have a large percentage of unfolded regions and no homology. Created with Biorender.com.

A new approach was designed to incorporate XL-MS restraints at each step of the modeling process (Fig. 1). It is designed to work on proteins that have low homology to existing structures and have XL-MS restraints in their flexible regions. AlphaLink, a modified version of OpenFold that incorporates experimental restraints in structural prediction, is used to generate initial models of the monomers (26).

The proteins are then brought together with MODELLER, a program that models target proteins based on templates by satisfying spatial restraints (36). This step includes the initial models built by AlphaLink and the intralinks present in the monomers to preserve their predicted structure while adjusting the structure to remove any distance violations, as well as the interlinks to bring the two proteins together.

This approach produces models that are consistent with experimental XL-MS data while also demonstrating good structure quality metrics.

### Cross-linking mass spectrometry of *Encephalitozoon hellem* reveals proximity interaction of spore wall proteins Swp1b and EnP1

We were interested in using this approach to model proteins of *Encephalitozoon hellem*. Three independent replicates of cross-linking mass spectrometry experiments were performed on spores of *E. hellem* employing Azide-A-DSBSO, a mass-spectrometer-cleavable cross-linker spanning 14 Å that can cross-link nearby lysine, serine, threonine, and tyrosine residues. Previous studies indicate that the maximum expected cross-linking distance cutoff between the C alpha atoms of cross-linked residues for this cross-linker is 30 Å, which incorporates the length of the cross-linker and amino acid side chains (37–39).

A total of 1,516 intralinks, 325 interlinks, and 112 selflinks (between the same amino acid residue on the same protein, indicating dimerization) were detected between the three replicates. Many proteins expected to interact with one another were cross-linked. For example, 35 interlinks were found between histone H3 and histone H4, 6 between histone H3 and histone 2B-like protein, and 3 between histone H4 and histone H2B-like protein. Sixteen links between 16 different ribosomal proteins (in both the large and small subunits) were found, and 3 links between 4 different proteasome subunits were found (Data S1).

**Fig 2:**
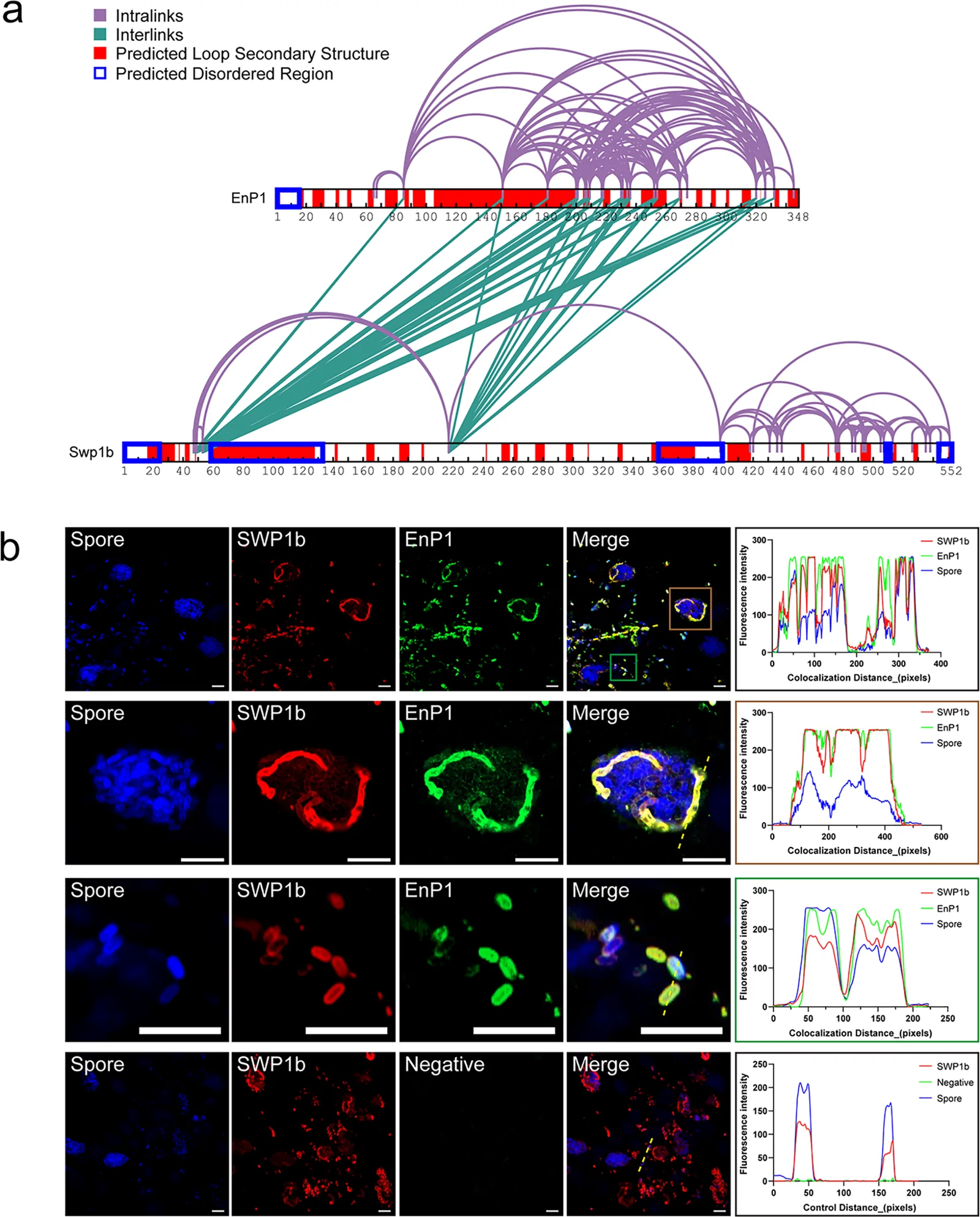
Swp1b and EnP1 are in close proximity to one another. (**a**) Intralinks (purple) and interlinks (green) mapped to amino acid sequences of Swp1b and EnP1. The location is indicated by the residue maps (Swp1b, 552 amino acids; EnP1, 348 amino acids). (**b**) Swp1b and EnP1 interact during spore maturation. The image shows spores within a parasitophorous vacuole inside an infected human foreskin fibroblast (HFF) host cell. Confocal imaging shows that Swp1b (red), EnP1 (green), and spores (blue, fluorescent brightener) exhibit co-localization at different developmental stages. ImageJ line-scan profiles (right) reveal synchronized fluorescence intensity peaks, with Pearson correlation coefficients consistently above 0.90, indicating a stable physical interaction between the two. Negative control: uninfected mouse serum. The yellow dashed line marks the analysis path in the merged image. Scale bar: 20 μm.

We identified 65 cross-links with medium (FDR ≤ 0.05) and high (FDR ≤ 0.01) confidence between Swp1b and EnP1, demonstrating that these proteins are in close proximity to one another. In addition, we identified 101 intralinks within EnP1 of medium and high confidence, and 58 intralinks within Swp1b of medium and high confidence (Fig. 2a; Data S2). Of these 224 cross-links, 97 were identical in at least two replicates (Fig. S1). EnP1 only had 8 links to 6 other proteins, and Swp1b had 11 links to 9 other proteins, leading us to be confident in the interaction between Swp1b and EnP1 (Data S1). These proteins are both highly abundant in the spore wall and are localized to two different layers of the spore wall, so we were interested in modeling their interaction as a potential drug target. To independently examine this protein-protein interaction, we performed co-localization analysis using anti-EnP1 and anti-Swp1b antibodies (Fig. 2b). Fluorescence co-localization analysis revealed co-localization of Swp1b and EnP1 throughout spore maturation. Confocal imaging demonstrated complete overlap of Swp1b and EnP1 at all developmental stages, from early to mature spores. The yellow dashed lines in the merged images indicate the analysis paths. Line-scan profiles along these paths, obtained using ImageJ, showed perfectly synchronized fluorescence intensity peaks across all three channels, with Pearson correlation coefficients consistently above 0.90 (mean *r* = 0.94 ± .03). Negative controls using pre-immune mouse serum confirmed the absence of non-specific signals, further supporting the XL-MS data, which indicate that these proteins are in close proximity.

### Deep learning alone does not accurately model the Swp1b-EnP1 complex

**Fig 3:**
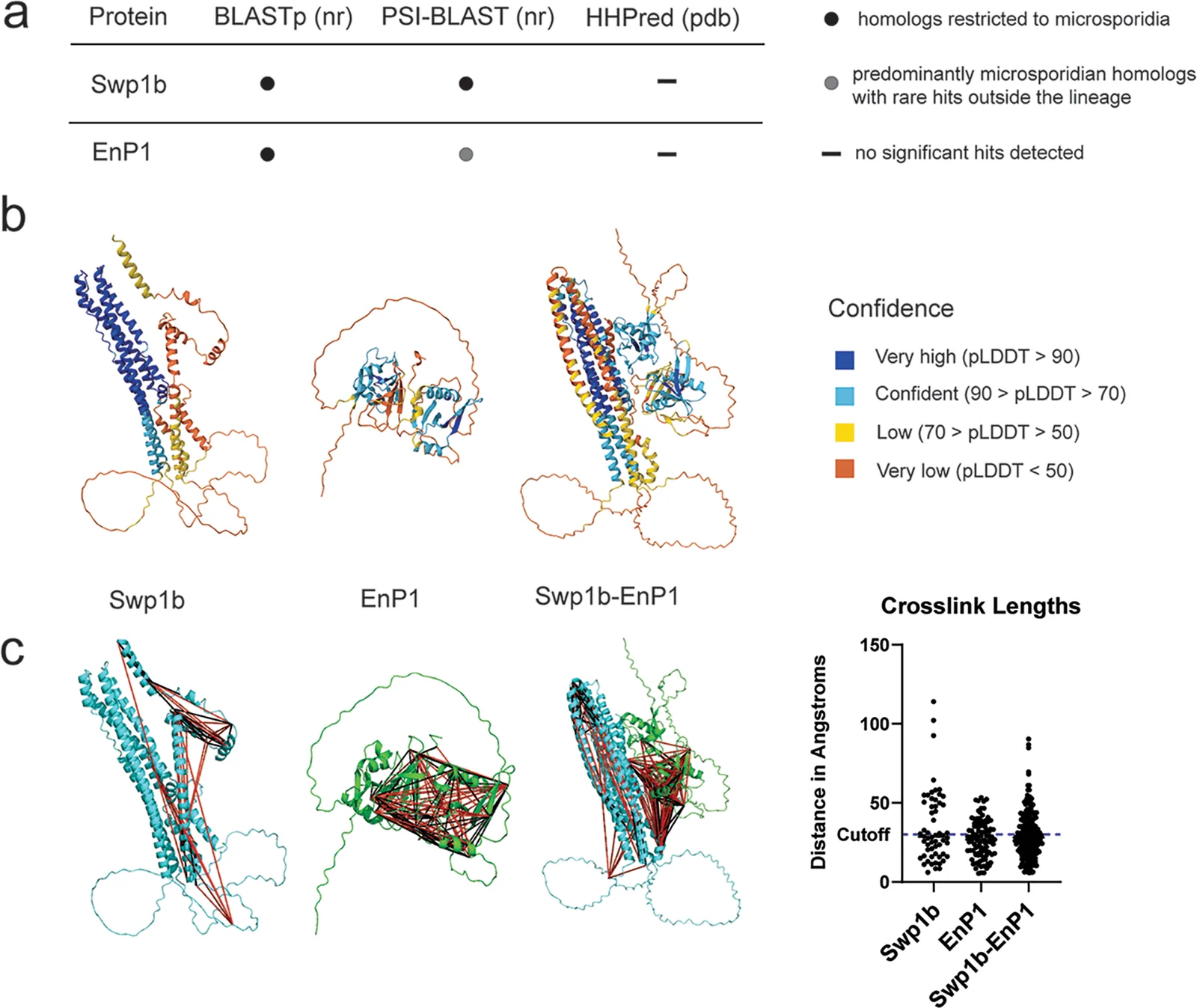
AlphaFold3 does not accurately predict the structure of Swp1b-EnP1. (**a**) Sequence similarity searches were performed using BLASTp and PSI-BLAST against the nr database, and structural homology was assessed using HHpred against PDB-derived databases. (**b**) AlphaFold3-predicted models colored by pLDDT. PAE plots are provided in Fig. S3. (**c**) AlphaFold3-predicted models with cross-links mapped onto the models with black lines. Distances are shown to the right, with the accepted cutoff of 30 Å marked with the dashed blue line. Cross-links with mapped distances above the 30 Å cutoff are marked in red on the models.

Swp1b and EnP1 are both major components in the spore wall, making them a desirable target to model their structures and interaction with each other to better understand the whole spore wall architecture. However, this is made difficult by the fact that both Swp1b and EnP1 are highly divergent proteins with sequence homology largely restricted to microsporidia. BLASTp found homologs for both proteins exclusively in microsporidia, while PSI-BLAST identified only a single probable bacterial homolog for EnP1. Neither protein showed significant similarity to proteins of known structure using HHpred (Fig. 3a). In addition, they both contain a significant portion of predicted flexible loop structure and disordered regions (Fig. 2a). AlphaFold has become the most used algorithm for predicting unknown protein structures; however, it must be used with caution. For example, in the case of Swp1b and EnP1, AlphaFold3 cannot predict the individual structures or the complex with confidence (pTM = 0.36, ipTM = 0.13). In addition to a low-confidence pLDDT score in several areas of the protein (Fig. 2b), the pTM and ipTM metrics, which measure the confidence in the overall predicted fold and position of the subunits, indicate a likely failed prediction. Typically, when using AlphaFold3, we are reliant on the confidence scores alone to evaluate the accuracy of the model. XL-MS cross-link data, however, provide experimental distance restraints that can be used to evaluate the accuracy of the AlphaFold3 models. When we map the distance restraints (Fig. 2a) to the AlphaFold3 structures, we see that this model fails to comply with 90/224 (40%) of the cross-links for the complex, indicating that the AlphaFold3 predicted model is not compatible with the experimental structural data (Fig. 3c). Even deep-learning algorithms like ESMFold that are designed to work on proteins with low homology do not produce structures compatible with our experimental distance restraints (Fig. S2).

### The AlphaLink-Modeller approach accurately models the Swp1b-EnP1 complex

AlphaLink, a modified version of OpenFold that incorporates experimental restraints in structural prediction, was used to generate initial models of Swp1b and EnP1. At this stage, 77/101 (76%) EnP1 intralink distances and 41/58 (71%) Swp1b intralink distances are satisfied, compared to 61/101 (60%) EnP1 intralink distances and 32/58 (55%) Swp1b intralink distances satisfied in the AlphaFold3 model.

Next, to enforce the set of 65 interlinks, the two proteins were brought together with MODELLER by adding the cross-links as additional distance restraints. This docked the two structures while preserving the global AlphaLink structures.

**Fig 4:**
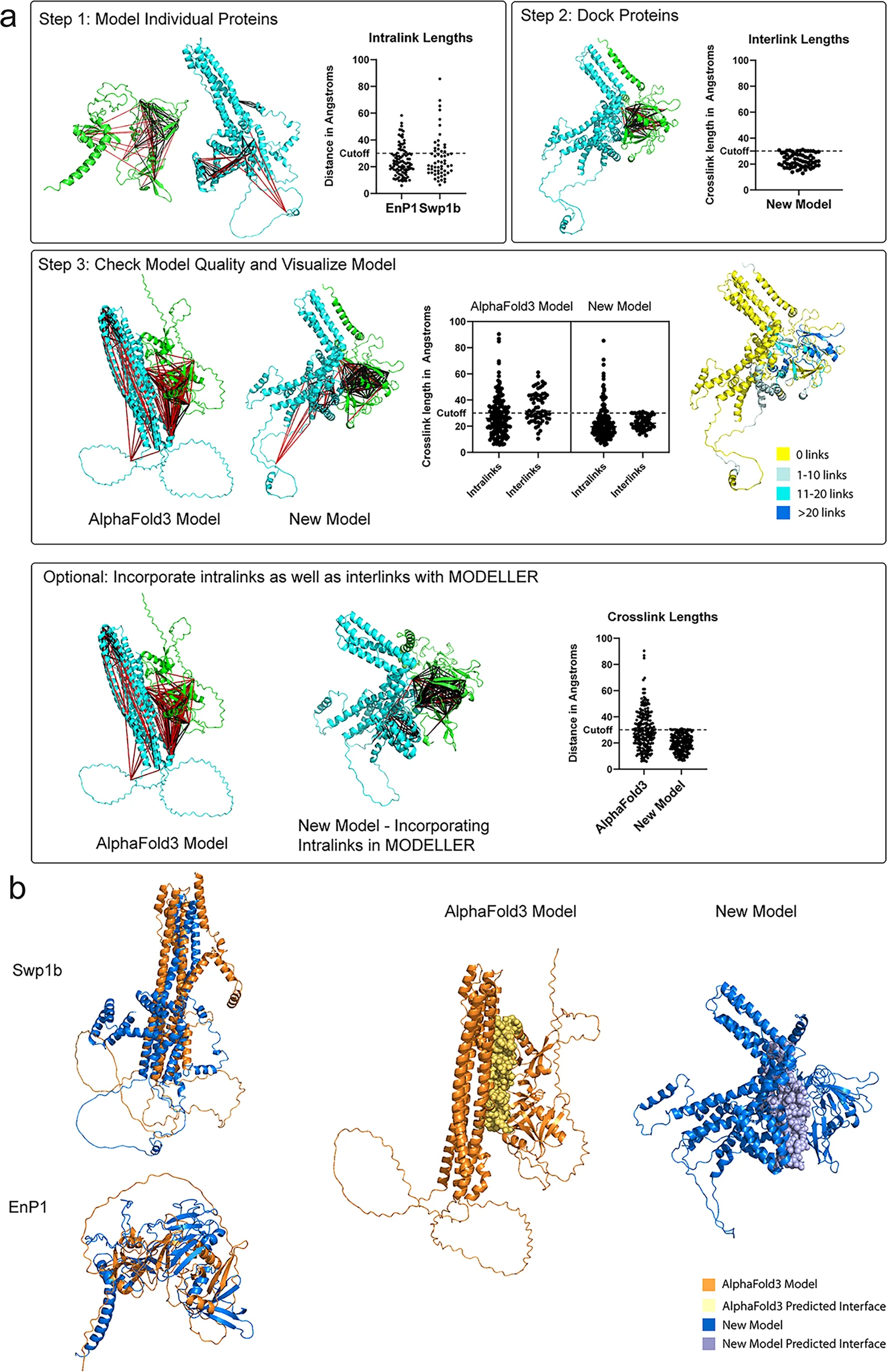
Stepwise modeling of Swp1b-EnP1 using the AlphaLink-Modeller approach. (**a**) In step 1, individual proteins are modeled with AlphaLink, and intralink distances are mapped on the right in black lines. Intralink distances violating the 30 Å cutoff are colored red. In step 2, the individual proteins are brought together with MODELLER, and the interlink distances are mapped on the right. In step 3, the model generated by AlphaFold3 (left) is compared to the model generated by our current methods (right). Cross-link lengths are mapped in the middle. To the right, the structure is colored by the number of cross-links in a 10-amino-acid window, with yellow being 0 links and dark blue more than 20. An optional addition to the methods is to incorporate intralinks along with the interlinks in Step 2. The resulting model is pictured on the left, and cross-link distances are mapped on the right. (**b**) Comparison of the AlphaFold3 model and the new model. On the left are individual models of Swp1b and EnP1 aligned. To the right are models of the complexes with residues within 4 Å of the other chain highlighted to represent the predicted interface.

When this approach is applied to the Swp1b-EnP1 complex, it produces a structure that is compatible with the experimental cross-linking data, with 55/65 interlink restraints (85%) falling under the 30 Å cutoff and the remaining 10/65 interlinks falling under 32 Å with an overall average length of 23.0 ± 5.1 Å. In addition, the structure contains most of its cross-links in the interface between Swp1b and EnP1, indicating the greatest confidence in that area (Fig. 4a). The model predicts five hydrogen bonds and eight salt bridges between Swp1b and EnP1 in the Swp1b-EnP1 interface (Fig. S4) (40). The model was also assessed for overall global and local model quality. Specifically, we calculated the RMS Z-score, which is a measure for assessing any inaccuracies in bond lengths and angles (41). The results show reasonable quantitative measures with a bond length score of 1.226, a bond angle score of 1.892, omega angle restraints of 1.314, and side chain planarity of 1.215, indicating no major deviations of bond and angular geometry from an optimized reference structure. Additionally, we assessed individual residue quality and composite model quality using the QMEAN4 score, which also shows reasonable model quality at the local residue level (Fig. S5) (42). Direct comparison with known available structures is difficult because the model and complex we propose are unique, but these scoring metrics provide reasonable model quality and validation. To further demonstrate the model quality and reproducibility, we performed an analysis where we randomly removed various numbers of interlinks and rebuilt the protein complex. When we did this, we saw that we can remove about 20 links before the structure significantly changes (Fig. S6), indicating a robust, reproducible outcome.

Optionally, MODELLER can also be used to resolve the remaining intralink distance violations by providing all intralinks as distance restraints along with the interlinks. This allows MODELLER to refine the individual structures produced by AlphaLink. When we perform the modeling this way, we get a structure with 208/224 cross-link restraints (93%) falling under the 30 Å cutoff and the remaining 16/224 cross-links falling under 31 Å with an overall average length of 19.9 ± 6.5 Å. Once again, we retain good model quality with a bond length score of RMS Z-score 1.166, bond angle score of 1.806, omega angle restraints of 1.487, and side chain planarity of 0.619.

When we compare the models to the one produced by AlphaFold3, which did not utilize cross-linking information, we see several differences. While the overall Swp1b model produced by AlphaLink is fairly similar, its C-terminal alpha helices are positioned above the N-terminal helix by AlphaFold3, whereas AlphaLink places them on the other side of the structure. In EnP1, we see a dramatic difference in the large loop in the middle of the structure. AlphaFold3 predicts a structure that has two lobes made of helices and beta sheets connected by a long, unstructured loop. Several cross-link restraints are present in that loop, so AlphaLink brings it in to interact with the other secondary structures, which AlphaLink predicts to have three lobes.

The complex predicted by the two methods is also quite different. They predict different interfaces between Swp1b and EnP1. Both AlphaFold3 and our method predict Swp1b residues around 215–260 to interact with EnP1, but ours predicts an additional area from approximately residues 440–500 also interacting with EnP1. Meanwhile, the two models predict completely different areas of EnP1 interacting with Swp1b. AlphaLink mostly predicts an area in residues 75–100 and 210–260 interacting with Swp1b. Our model predicts an area in residues 20–60 and 270–350 interacting with Swp1b. Since our model better fulfills the experimental distance restraints between these two proteins, we are confident that our predicted interface is more accurate (Fig. 4b).

**Fig 5:**
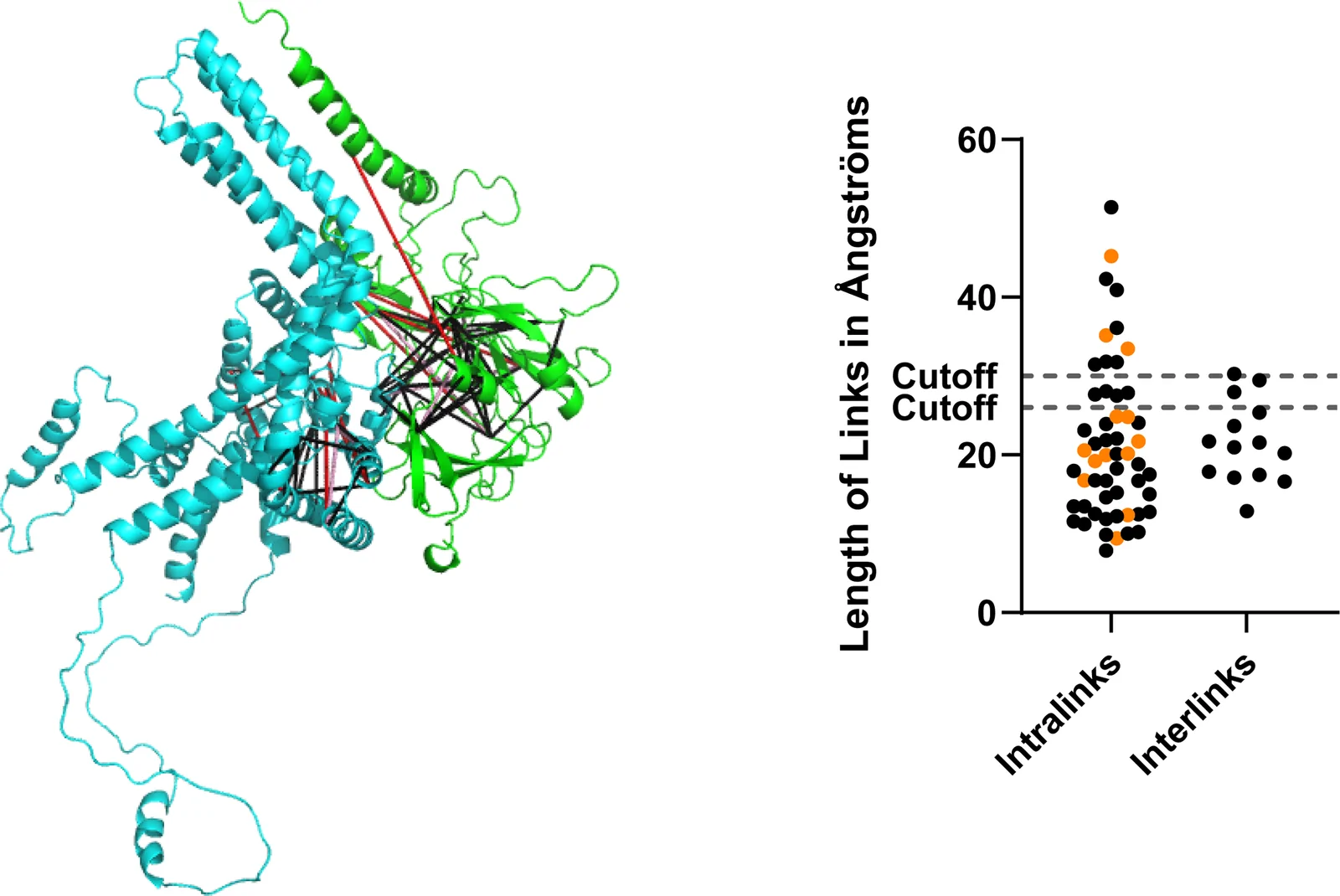
Independent data set maps onto the Swp1b-EnP1 model. Cross-links generated from an independent DSSO run were mapped onto the Swp1b-EnP1 model with links violating the 30 Å cutoff colored red (left), links violating the 26 Å cutoff colored pink (left), and cross-link length distribution from the independent run (right). Newly discovered links are colored orange on the right.

Our confidence in the AlphaLink-Modeller model was further tested as follows. The models were generated with cross-links combined from three separate XL-MS runs. As an additional test of the resulting model, we performed a fourth, small-scale XL-MS run completely independent of the first three and identified a total of 68 intralinks and interlinks in Swp1b-EnP1. This run was performed with DSSO, a different cross-linker that, according to the literature, has a distance cutoff of 30 Å. However, when we calculate the maximum distance for the 10.1 Å DSSO linker using the same methodology as Azide-A-DSBSO, we end up with a distance cutoff of 26 Å. Of these links, 51/68 (75%) fell under 26 Å, 57/88 (84%) under the theoretical cutoff of 30 Å, with four additional links within only 1 Å violation of the cutoff. Links were also present at 33, 35, 36, 40, 42, 45, and 51 Å. Of these DSSO links, 55 were identical to previously observed links and 13 were new links. Of the 55 identical links, 41 were within 26 Å, 47 were within 30 Å, and 4 were within 32 Å, with the rest being 36, 40, 42, and 51 Å. These are intralinks that AlphaLink was unable to bring under the distance restraint. Of the 13 novel links, 10 were within 26 Å, 1 was 33 Å, 1 was 35 Å, and 1 was 45 Å (Fig. 5; Data S3). The link measuring 45 Å is an EnP1 intralink. One end of the link is in an alpha helix with no other cross-links having been previously identified in this area. Independently generated data mapping well to our generated model gives us confidence in the accuracy of our complex prediction.

## DISCUSSION

In this paper, we use a new integrative modeling approach that incorporates XL-MS restraints into structure prediction of low-homology, flexible proteins. We used this pipeline to model a previously unidentified interaction between Swp1b and EnP1. While default deep-learning models failed to satisfy experimental XL-MS restraints, our integrative modeling approach produces models consistent with our experimental data.

Deep-learning protein prediction algorithms excel at predicting proteins with structural motifs already observed before (43). But they struggle more when faced with proteins with unique sequences and flexible regions. Most deep-learning algorithms are trained to use multiple sequence alignments. Proteins without any detectable homologs lack this information, and as a result, structures predicted by AlphaFold3 and similar algorithms are low confidence. Even deep-learning algorithms that do not rely on multiple sequence alignments still struggle with low-homology proteins, as they rely on sequence patterns as opposed to physical and chemical properties. As a result, predictions for low-homology proteins are often of low confidence. In these cases, additional experimental data are needed to guide and evaluate modeling. Orthogonal experimental data such as XL-MS provide this type of experimental data to guide accurate structure prediction.

**Fig 6:**
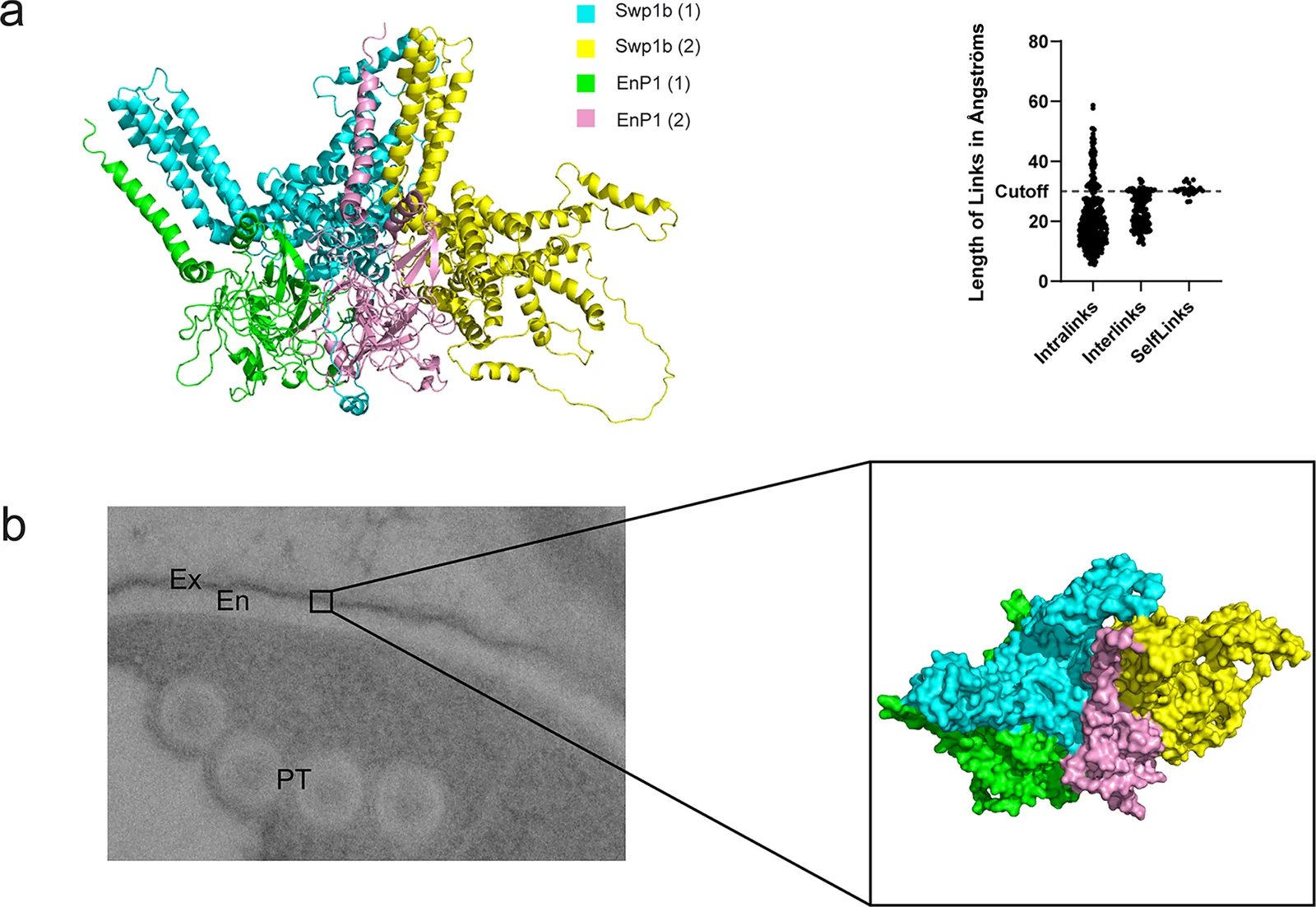
Model of the interface between the endospore and exospore. (**a**) Model of the Swp1b-EnP1 heterotetramer. (left) with cross-link distances mapped on the right. (**b**) TEM image of the spore wall. Ex, exospore; En, endospore; PT, polar tube. The predicted model of Swp1b subunits (blue and yellow) and EnP1 subunits (green and pink) forms a tight interface that we hypothesize is located at the interface between the exospore and endospore.

Modeling of Swp1b and EnP1 provides biological insight into microsporidia spore wall architecture. Prior transmission electron microscopy experiments localized Swp1b to the exospore and EnP1 to the endospore. Due to their location in separate layers of the spore wall, the modeling of this interface between the proteins can reflect the structure of the interface between these two layers. Links were found between the same amino acid residue of the same protein (self-links), indicating the presence of homodimers. By incorporating these links and bringing two heterodimers together, we can model what a tetramer in the exospore-endospore interface may look like (Fig. 6). It also demonstrates an example of flexible proteins becoming ordered during assembly. This observation supports the broader concept that flexible regions can become structured in specific contexts, such as binding with a cognate partner. We believe this to be the case in Swp1b and EnP1 because, individually, they are predicted to have large regions of disorder, and we were not able to detect this interaction using yeast-two-hybrid or co-IP experiments (data not shown, Fig. S7 and S8) when these proteins were expressed in the cytoplasm of yeast, *Escherichia coli,* or mammalian cells (HEK1). This argument is supported by cryo-EM images of the spore wall showing that it is highly organized, supporting regularly folded proteins instead of disordered proteins (44). This suggests that these proteins must be in the spore wall environment to fold correctly. This could be due to a number of factors, e.g., there are other spore wall proteins present in the exospore and endospore that may contribute to their folding correctly, and the endospore is largely made of chitin, which could act as a scaffold and influence EnP1’s structure in the spore wall. When modeling the two proteins together, we can see they form a tight interface (Fig. 6). We believe that, due to the abundance of these proteins and the location of their interaction, this complex is important for creating environmentally resistant spore walls. There are no genetic systems for microsporidia (i.e., no transfection systems or methods to modify genes within these organisms have been developed), so we are not able to generate or test these models by altering genes within *E. hellem*. As this interaction is potentially important for the spore wall, our current model might serve as a starting point for drug development studies to disrupt this interaction.

It is important to note the limitations of this approach. For example, this pipeline may not work for all disordered proteins. The approach is designed to identify a mostly ordered state, whether that is due to environmental context or binding with a partner.

This approach also does not intrinsically know the stoichiometry of an assembly. AI models tend to be able to better predict assemblies when the stoichiometry is known, which must be clarified with other experimental methods. This model assumes a 1:1 Swp1b-EnP1 complex, but XL-MS alone does not establish stoichiometry. It is possible that alternative assembly states are present in the spore wall and that the individual proteins may adopt multiple conformations depending on their binding partners and location in the spore wall layer.

The results presented illuminate the value of integrative approaches that combine AI-based and physics-based predictions with experimental data such as XL-MS for modeling proteins that are experimentally inaccessible and can be predicted with low confidence only using default approaches. As structural biology moves toward defining protein machinery by the incorporation of “omics”-level data, the use of this type of analytical pathway that can use such data to refine structural predictions is obvious and will be an essential part of computational modeling that aims to derive biologically meaningful structures.

## MATERIALS AND METHODS

### Cell culture

*E. hellem* was grown in human foreskin fibroblast cells (HFFs; ATCC: CRL-1634; Hs27) in Dulbecco’s modified Eagle medium supplemented with fetal calf serum, L-glutamine, and penicillin-streptomycin at 37°C and 5% CO_2_. Spores were passaged to new HFFs once a week. The spores in the supernatant were then collected after lysing out of the HFFs. After spinning down at 2,000 × *g* for 20 min, they were then resuspended in 30 mL PBS and put through a 27-gauge needle thrice and filtered through a 5-μm filter (Millipore) once. They were then centrifuged at 2,000 × *g* for 20 min and resuspended in 15 mL 1% H_2_O_2_ in PBS. They were then incubated at 37°C rotating overnight to allow them to germinate. The spores were then centrifuged at 2,000 × *g* for 20 min and washed three times with distilled water.

### Cross-linking mass spectrometry of *Encephalitozoon hellem*

The cross-linking protocol was performed following our previously published method (45).

*E. hellem* spores were germinated with 1% H_2_O_2_ in 1× PBS rotating at 37°C overnight. The approximate protein concentration was measured with a Bradford assay, and the protein concentration was adjusted to approximately 1 μg/μL. Azide-A-DSBSO was slowly added to the solution to a final concentration of 5 mM, followed by incubation for 2 h at room temperature with rotation. Cross-linking was quenched by adding Tris-HCl pH 8 at 50 mM and incubation for 5 min at room temperature. Spores were pelleted and washed three times with 50 mM Tris-HCl pH 8.

The pellets were resuspended in 5% sodium deoxycholate (SDC), 5% acetonitrile, and 130 mM TCEP in 50 mM ABC and rocked at room temperature for 30 min. IAA was added to 260 mM and incubated at 60°C in the dark for 30 min. The solution was spun at maximum speed for 5 min to separate the soluble and insoluble material. The soluble material was loaded onto a 30 kDa Amicon Ultra filter device (Millipore Sigma) and washed with 10% acetonitrile in 50 mM Tris-HCl pH 8 to remove the SDC. The insoluble fraction was pelleted and washed three times to remove the SDC. LysC 1:25 was added to either the filter or directly to the tube and incubated overnight at 37°C. The next day, trypsin was added at 1:10 and CaCl_2_ was added to a final concentration of 10 mM and incubated at 47°C for 1 h. The Amicon filter was spun down and washed with 50% acetonitrile in 50 mM Tris-HCl pH 8 to collect the peptides. The insoluble material was spun at maximum speed, and the supernatant was kept.

After digestion, acetonitrile concentration was adjusted to 10% and incubated with DBCO beads at a concentration of 20 μL per 1 mg of peptide for 1 h at room temperature with rotation. The DBCO beads were washed three times with 1 M NaCl and 10% acetonitrile in 25 mM ABC. The peptides were eluted with 10% trifluoroacetic acid (TFA) by rotating the tubes at room temperature for 30 min. The beads were eluted two times, and the ABC was added to a final concentration of 100 mM. The beads were then eluted with 50% acetonitrile in 25 mM ABC two times. All eluates were combined and vacuum dried. Samples were desalted with Pierce C18 spin columns according to the manufacturer’s protocol, with an additional wash step of 5% acetonitrile and an additional elution step of 90% acetonitrile.

However, as the cross-linking protocol for *E. hellem* was being optimized at the time, all three runs had slightly different parameters. Changes were as follows. In run 1, five 150 mM plates of HFFs were used. The cross-linker was incubated for 4 h at room temperature. Proteins were reduced with 130 mM TCEP, alkylated with 260 mM IAA, and digested with 1:25 trypsin over 24 h. Next, 20 µL of DBCO beads were added per mg of initial protein. No high pH fractionation was performed. In run 2, 15 plates of HFFs (150 mM) were used. The cross-linker was incubated for 1 h at room temperature. Proteins were reduced with 130 mM DTT in 5% sodium deoxycholate, alkylated with 260 mM IAA, digested with 1:25 LysC at 37°C overnight, then digested with 1:10 trypsin at 47°C for 1 h. DBCO beads, 20 μL per 1 mg measured peptide concentration, were added. No high pH fractionation was performed. Run 3 had the same parameters as run 2 except high pH fractionation was performed as previously described by adding 5%, 10%, 15%, 20%, 25%, 30%, 35%, and 50% acetonitrile sequentially, and two fractions were combined (5% + 25%, 10% + 30%, 15% + 35%, and 20% + 50%) (45).

Run 4 was performed as a test of a new method. Germinated spores were cross-linked with 1 mM DSSO for 30 min with shaking at room temperature. Proteins were reduced with 130 mM TCEP at 70°C for 1 h and alkylated with 260 mM IAA for 30 min at room temperature. Phosphoric acid to 1.2% (vol/vol) was added to the solution, and the sample was loaded onto a midi S-trap binding column. It was washed with S-trap binding buffer, and LysC was added at 1:25 and digested for 4 h at 37°C. Trypsin was added at 1:25, and digestion was performed overnight at 37°C. Peptides were eluted as per S-trap instructions, with an extra elution step of 90% acetonitrile and 0.1% TFA, and peptides were dried in a speed-vac. The peptides were then resuspended to 10 mg/mL in 30% acetonitrile, 0.1% TFA. A total of 1.7 mg of peptide was injected into a Superdex 30 Increase 10/300 column (Cytiva), run with a 0.5 mL/min flow rate, and eluted isostatically with the same buffer. Fractions of 100 µL were collected. Fractions from the first six peaks were selected and dried.

### Mass spectrometric analysis

Mass spectrometric analysis for all runs was performed as previously described (45). Samples were resuspended in 0.1% TFA and loaded onto a Dionex RSLC Ultimate 3000 (Thermo Scientific), coupled online with an Orbitrap Exploris 480 (Thermo Scientific) with FAIMS Pro attached. Chromatographic separation was performed with a PicoFrit analytical column (75 µm ID, 25 cm length) packed in-house with reversed-phase Repro-Sil Pur C18-AQ 3 µm resin. Peptides were separated using a 180-min gradient from 4% to 30% buffer B (buffer A: 0.1% formic acid; buffer B: 80% acetonitrile + 0.1% formic acid) at a flow rate of 300 nL/min. The mass spectrometer was set to acquire spectra in a data-dependent acquisition mode. Briefly, the full MS scan was set to 375–1600 m/z with a resolution of 120,000 (at 100 *m*/*z*). MS/MS was performed at 60,000 resolution and an AGC target of 1 × 10^4^ and stepped HCD normalized collision energy of 19%, 25%, and 32% with FAIMS compensation voltages of −50, −60, and −75 (46–49).

The data were analyzed with Proteome Discoverer 2.5 with IMP-MS2 spectrum processor for deisotoping and MS Amanda 2.0 with the following parameters: maximum missed cleavage 4, MS1 tolerance 10 ppm, MS2 tolerance 20 ppm, static modification: carbamidomethyl 57.021 Da (C), and dynamic modifications: oxidation 15.995 Da (M), DSBSO amidated 325.065 Da (K), and DSBSO hydrolyzed 326.049 Da (K). Spectrum searches were performed on MicroporidiaDB (microsporidiadb.org) release 65 (50). Data sets were analyzed with MS Annika 2.0 as MS-cleavable cross-linkers with cross-link doublet 308.039 on K, S, T, and Y residues (51).

The fourth data set was analyzed with MS Amanda 2.0 with parameters DSSO Amidated 175.030 Da (K) and DSSO Hydrolyzed 176.014 Da (K). It was analyzed with MS Annika 2.0 as MS-cleavable cross-linkers with a cross-link doublet 158.004 on K, S, T, and Y residues.

### Data processing

The data generated by Annika 2.0 were processed using R with tidyverse packages. FDR was recalculated using a custom R script (45). Peptide pairs are collapsed to residue-to-residue pairs. Quality scores are adjusted from lower-level scores by calculating Euclidean norms, and the FDRs were recalculated at higher levels after inter/intra cross-links were separated according to the previously described method (52). Cross-links were combined from all three runs. Cross-links appearing in multiple runs were weighted accordingly (cross-links found in two runs occurred two times; cross-links found in three runs occurred three times). When analyzing distances, only unique cross-links were calculated. Links between Swp1b and EnP1 were visualized with xiVIEW (https://xiview.org/index.php) (53). Secondary structure prediction was performed with PSIPRED (https://bioinf.cs.ucl.ac.uk/psipred/).

### Immunofluorescence staining of Swp1b and EnP1

HFF (ATCC CRL-2522) cells and *E. hellem* were maintained in DMEM (Gibco) supplemented with 10% fetal bovine serum (FBS) and 1% penicillin-streptomycin (Gibco) at 37°C under 5% CO_2_. For experiments, cells were seeded at 5 × 10^⁴^ cells/well on glass coverslips in 24-well plates. Fixed with 4% paraformaldehyde (PFA) for 15 min at room temperature (RT), cells were permeabilized with 0.1% Triton X-100 (15 min, RT) and blocked with 5% bovine serum albumin (BSA; Sigma-Aldrich, A7906; 1 h, RT). Primary antibodies were applied overnight at 4°C, followed by incubation with Alexa Fluor 488-conjugated goat anti-mouse IgG (1:2000; Thermo Fisher Scientific, A32723) or Cy3-labeled goat anti-rat IgG (H + L) (1:500; Beyotime, A0507) for 1 h at RT in the dark. Nuclei were counterstained with fluorescent brightener (Sigma, MKCL1227) for 5 min. Coverslips were mounted with antifade medium (Solabio) and imaged using a Zeiss LSM 980 confocal microscope (63× oil objective) with Z-stack acquisition (0.5 μm intervals). Maximum intensity projections were generated using ZEN Blue software. Fluorescence intensity was quantified in ImageJ with consistent background subtraction and thresholding across samples. Primary antibodies: anti-EnP1 mouse (1:200); anti-SWP1b rat (1:200).

### Sequence and structural homology analysis and secondary structure analysis

Sequence similarity searches were performed using BLASTp and PSI-BLAST (https://blast.ncbi.nlm.nih.gov/Blast.cgi) against the clustered non-redundant (nr) database (54). Significant hits (*E* ≤ 1 × 10^−^⁵) were used to assess the taxonomic distribution of homologs. Structural homology was evaluated using HHpred (https://toolkit.tuebingen.mpg.de/tools/hhpred) against PDB-derived databases (55). Reciprocal BLASTp searches were performed for candidate remote homologs.

Secondary structure analysis was performed with PSIPRED (https://bioinf.cs.ucl.ac.uk/psipred/). PSIPRED 4.0 was used to predict the secondary structure, and DISOPRED3 was used to predict disordered regions using a cutoff of 0.5 (56, 57).

### AlphaFold3 modeling

Individual proteins and the Swp1b-EnP1 multimer were modeled with the AlphaFold3 server (https://alphafoldserver.com/) with standard settings (4).

### Integrative structural modeling

After cross-links were extracted with RRI_FDR.R, intralinks were extracted with getAlphaLinkFile.R. All intralinks with an FDR ≤ 0.05 were included. Initial structural models of Swp1b and EnP1 were generated using AlphaLink, a modified version of OpenFold that incorporates experimental distance restraints into structural prediction (26). Intralinks were provided as distance distributions generated by AlphaLink. An upper bound distance restraint of 30 Å was provided. For each protein, the relaxed output model was selected for downstream analysis.

The EnP1 model was modified so it was labeled as chain “B” and transformed 150 Å in the x direction by modify_pdb.R to clearly separate them from one another. The two protein monomers were used as templates and brought together using MODELLER (https://salilab.org/modeller/), incorporating all interlinks with FDR ≤ 0.05 as 30 Å upper-bound distance restraints with a standard deviation of 0.1 by redefining the AutoModel.special_restraints() routine as described in the MODELLER manual (36). Models were deposited in PDB-IHM (https://data.pdb-ihm.org/).

All scripts used and an example .pir alignment file are provided in supplemental materials.

Cross-links are mapped onto the final model, and their distances are measured with calculateMultimerDistance.py by creating distance objects in PyMOL (58). RMS Z-scores were obtained from the WHATCHECK tool (https://bio.tools/what_check) with default settings (41). QMEAN (available via SWISSMODEL) (https://swissmodel.expasy.org/qmean/) score was generated to assess the confidence for each residue (42). iCn3D was utilized to analyze the number of hydrogen bonds and salt bridges (https://www.ncbi.nlm.nih.gov/Structure/icn3d/) (59, 60).

### ESMFold modeling

ESMFold was accessed through Neurosnap (https://neurosnap.ai/login). Six recycles were performed for EnP1, and 15 recycles were performed for Swp1b and Swp1b-EnP1. Structures were analyzed in PyMOL.

### RMSD analysis

Three sets of interlinks were randomly selected to be removed from the analysis. Using the AlphaLink-generated models, the complex was modeled as previously described with MODELLER. RMSD was calculated with the MDAnalysis Python library by aligning both individual chains to calculate the RMSD of each chain, then performing global alignment and measuring the global RMSD. The script is provided in the Supplemental material.

### Scripts and files

RRI_FDR.R extracts cross-link data from Annika2.0. getAlphaLinkFile.R extracts intralinks for proteins of interest and puts them in AlphaLink format. modify_pdb.R modifies a pdb file for MODELLER format. xl-for-modeller.R exports cross-links in MODELLER format.

xl-for-modeller-plus-intralinks.R exports cross-links and intralinks in MODELLER format. Swp1b-EnP1.pir Example .pir file for bringing together two chains in MODELLER.

model_w_restraints.py is an example file to run MODELLER. calculateMultimerDistance.py maps and records cross-link distances in PyMOL. All scripts are provided in the Supplemental material and have been deposited on GitHub (https://github.com/eweyer/XL-MS-Guided-Structure-Prediction-of-Disordered-Encephalitozoon-hellem-Proteins).

### Yeast two-hybrid assay

Coding regions for Swp1b were amplified from *E. hellem* DNA using the following primers for each construct: Swp1b-FL-forward (TGATCTCAGAGGAGGACCTGGGAGAGAGAAGGAGAAG), Swp1b-FL-reverse (CCAAGGGGTTATGCTAGTTATTATTCCTTTTCTTCATATGC), Swp1b^32-352^-forward (TGATCTCAGAGGAGGACCTGGCTAATGGCTCCAGGTATTTC), Swp1b^32-352^-reverse (CCAAGGGGTTATGCTAGTTAGAAAATCCTGGCCGCAAG), Swp1b^20-60^-forward (TGATCTCAGAGGAGGACCTGGGAGAGAGAAGGAGAAGCGG), Swp1b^20-60^-reverse (CCAAGGGGTTATGCTAGTTAGGGCCTTTCTCCACAAAGCC), (GGGGS)_3_-Swp1b-forward (TGATCTCAGAGGAGGACCTGGGAGGAGGAGGATC TGGAGGAGGAGGATCTGGAGGAGGAGGATCTGGAGAGAGAAGGAGAAG), and (GGGGS)_3_-Swp1b-reverse (CCAAGGGGTTATGCTAGTTATTATTCCTTTTCTTCATATGC).

Coding regions for EnP1 were amplified from *E. hellem* DNA using the following primers for each construct: EnP1-FL-forward (ACGACGTACCAGATTACGCTAAGACC AAGGCTCTTCATC), EnP1-FL-reverse (TGGCGAAGAAGTCCAAAGCTTTACAATCGGCT GTAATCC), EnP1^141-348^-forward (ACGACGTACCAGATTACGCTTACGTCCAAA GAGAAGAGAAC), EnP1^141-348^-reverse (TGGCGAAGAAGTCCAAAGCTT TACAATCGGCTGTAATCCAC), EnP1^141-215^-forward (ACGACGTACCAGATTACGCTTACGTCCAAAGAGAAGAGAAC), EnP1^141-215^-reverse (TGGCGAAGAAGTCCAAAGCTTTAAACGTCAAATGTGAATGTC), (GGGGS)_3_-EnP1-forward (ACGACGTACCAGATTACGCTGGAGGAGGAGGATCTGGAGGAG GAGGATCTGGAGGAGGAGGATCTAAGACCAAGGCTCTTCATC), and (GGGGS)_3_-EnP1-reverse (TGGCGAAGAAGTCCAAAGCTTTACAATCGGCTGTAATCC).

Following amplification, each PCR product was inserted into the corresponding sites in the yeast two-hybrid vectors pGBKT7 and pGADT7 (Matchmaker Gold Yeast Two-Hybrid System, Takara Bio USA) with NEBuilder HiFi DNA Assembly. The identity of all clones was confirmed by sequencing the completed constructs.

For interaction studies, Y2HGold yeast strain was transformed as per the manufacturer’s instructions with the Yeastmaker Yeast Transformation System 2 (Takara Bio, USA) and plated onto 100-mm SD-selective plates. The plates were incubated at 30°C for 3 days, and results were recorded.

### Co-immunoprecipitation assay

HEK293T (ATCC CRL-11268) cells were transiently transfected with the indicated plasmids (pcDNA-SWP1b-3HA and pcDNA-EnP1-Flag) using Lipofectamine 3000 transfection reagent (Thermo Fisher Scientific) according to the manufacturer’s instructions. Forty-eight hours post-transfection, cells were harvested and whole-cell lysates were prepared. For immunoprecipitation, lysates were incubated with anti-HA magnetic beads (MCE, HY-K0201) or anti-Flag magnetic beads (MCE, HY-K0207) at room temperature for 1 h with gentle rotation. A small aliquot of the input lysate was reserved as an input control. Following incubation, the beads were washed, and bound proteins were eluted and denatured in SDS loading buffer. Samples were then resolved by SDS-PAGE and transferred to membranes for western blotting. Primary antibodies against the respective tags were used at a dilution of 1:2,000 (HA-Tag mAb, Abmart, 26D11; DYKDDDDK-Tag mAb, Abmart, M20008S). Protein bands were visualized using enhanced chemiluminescence (ECL; Millipore) and imaged on a Tanon-5200 imaging system. Densitometric quantification of band intensities was performed using ImageJ software.

## SUPPLEMENTAL MATERIAL

Data S1 mbio.02261-26-s0001.xlsx *E. hellem* crosslinks.

Data S2 mbio.02261-26-s0002.xlsx Swp1b-EnP1 crosslinks.

Data S3 mbio.02261-26-s0003.xlsx Independent set of crosslinks.

Supplemental Figures mbio.02261-26-s0004.pdf Figures S1 to S8.

Supplemental Legends mbio.02261-26-s0005.docx Supplemental figure, data, and script legends.

Supplemental Material mbio.02261-26-s0006.txt Scripts.

ASM does not own the copyrights to Supplemental Material that may be linked to, or accessed through, an article. The authors have granted ASM a non-exclusive, world-wide license to publish the Supplemental Material files. Please contact the corresponding author directly for reuse.
