# Supplementary material for: Cross-linking mass spectrometry guided structure prediction of disordered Encephalitozoon hellem proteins
Source: mBio. 2026 Oct 20;17(11):e02261-26. doi: 10.1128/mbio.02261-26 (PMC13753748; doi:10.1128/mbio.02261-26)
Supplement: Supplementary file 1 [file mbio.02261-26-s0004.pdf]

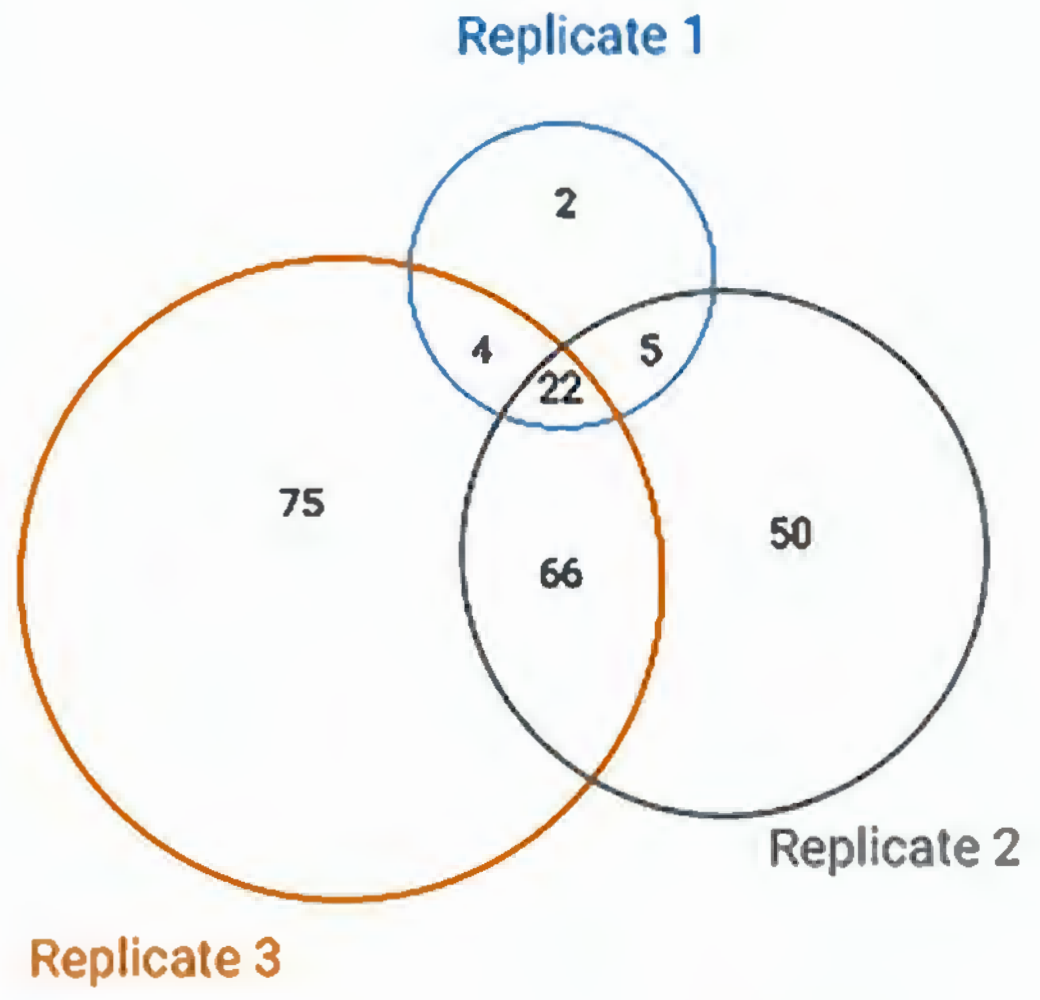
Replicate 1
2
4
5
22
75
66
50
Replicate 2
Replicate 3

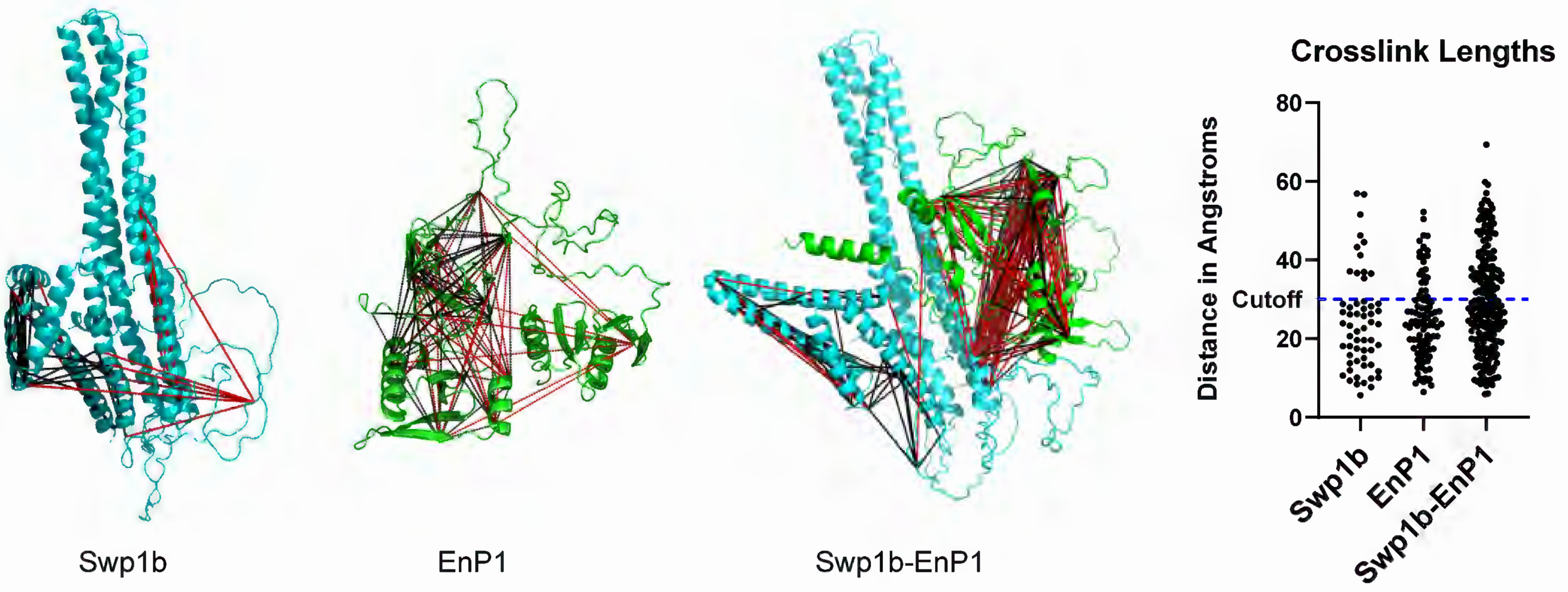
Swp1b
EnP1
Swp1b-EnP1
Crosslink Lengths
Distance in Angstroms
80
60
40
Cutoff
20
0
Swp1b
EnP1
Swp1b-EnP1

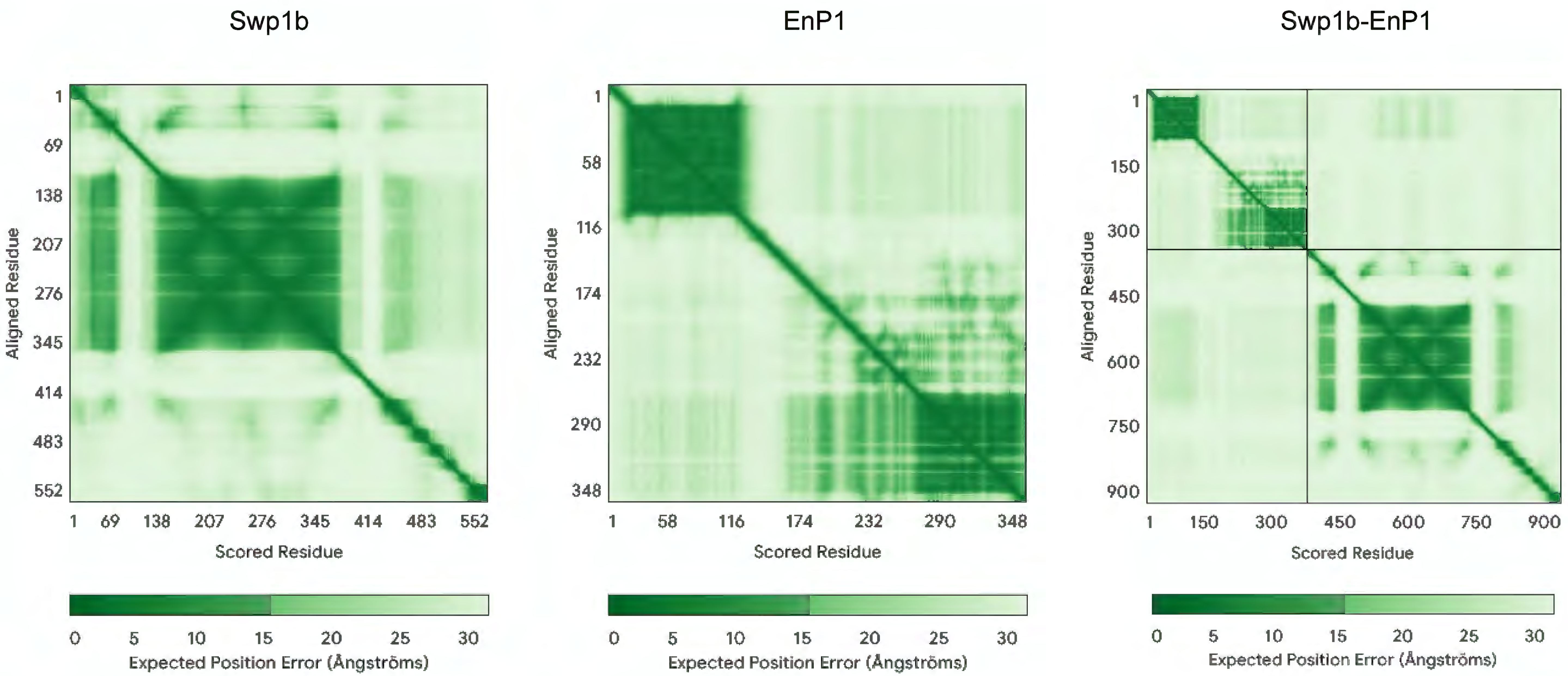
Swp1b
EnP1
Swp1b-EnP1
Aligned Residue
Scored Residue
1
69
138
207
276
345
414
483
552
58
116
174
232
290
348
150
300
450
600
750
900
0
5
10
15
20
25
30
Expected Position Error (Ångströms)

H-bonds (total = 5)
Swp1b_ASP_50 (O) – EnP1_ARG_276 (N)
Swp1b_SER_248 (O) – EnP1_HIS_52 (N)
Swp1b_SER_252 (OG) – EnP1_HIS_52 (O)
Swp1b_GLN_311 (NE2) – EnP1_ASP_49 (O)
Swp1b_GLU_463 (O) – EnP1_ASN_86 (ND2)
**Salt Bridges (total = 8)**
Swp1b_ASP_50 (OD1) – EnP1_HIS_74 (ND1)
Swp1b_ASP_50 (OD1) – EnP1_HIS_74 (NE2)
Swp1b_ASP_50 (OD1) – EnP1_LYS_274 (NZ)
Swp1b_ASP_50 (OD1) – EnP1_ARG_275 (NH1)
Swp1b_LYS_217 (NZ) – EnP1_ASP_73 (OD1)
Swp1b_GLU_471 (OE1) – EnP1_HIS_84 (NE2)
Swp1b_LYS_495 (NZ) – EnP1_ASP_301 (OD1)
Swp1b_LYS_505 (NZ) – EnP1_GLU_303 (OE1)

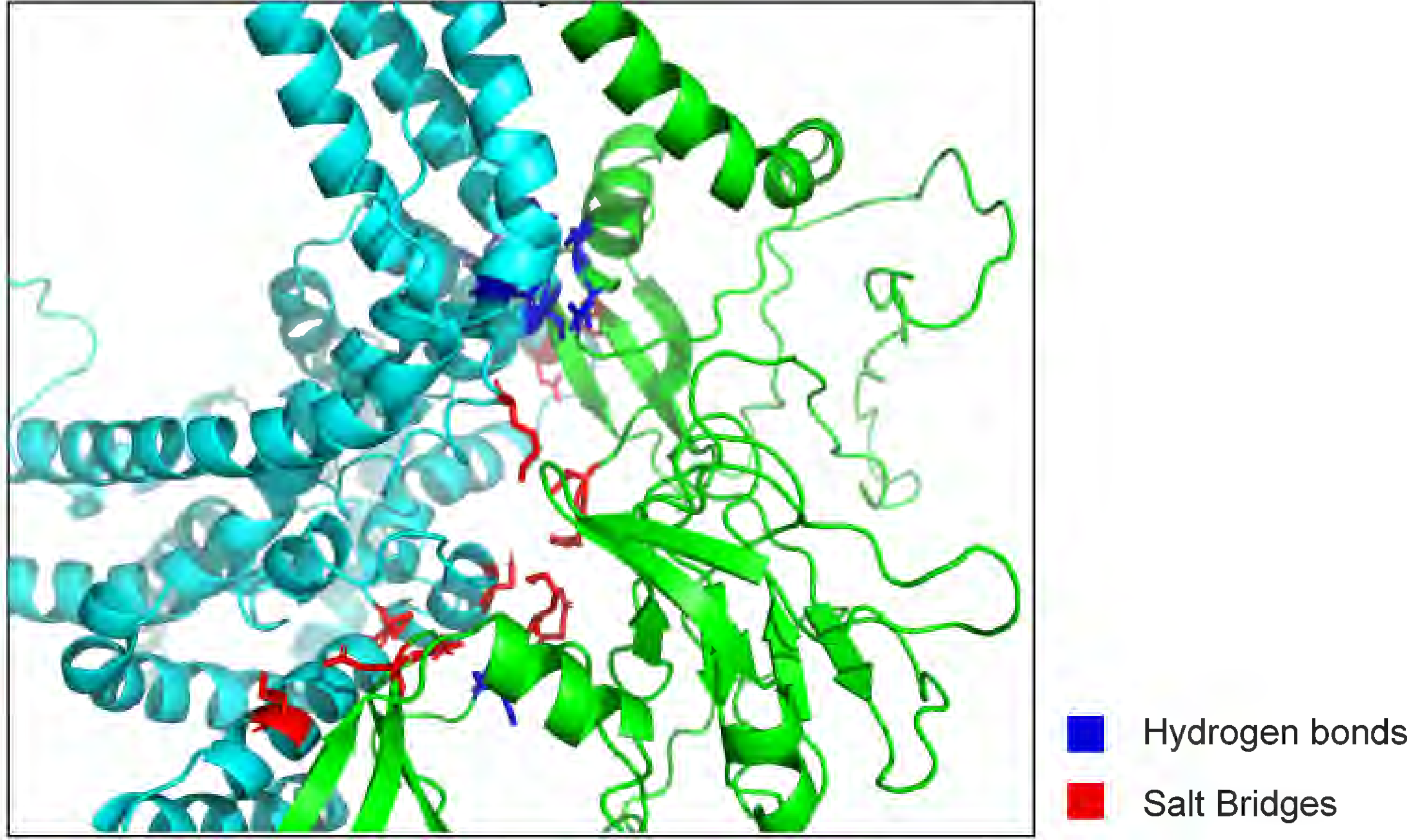

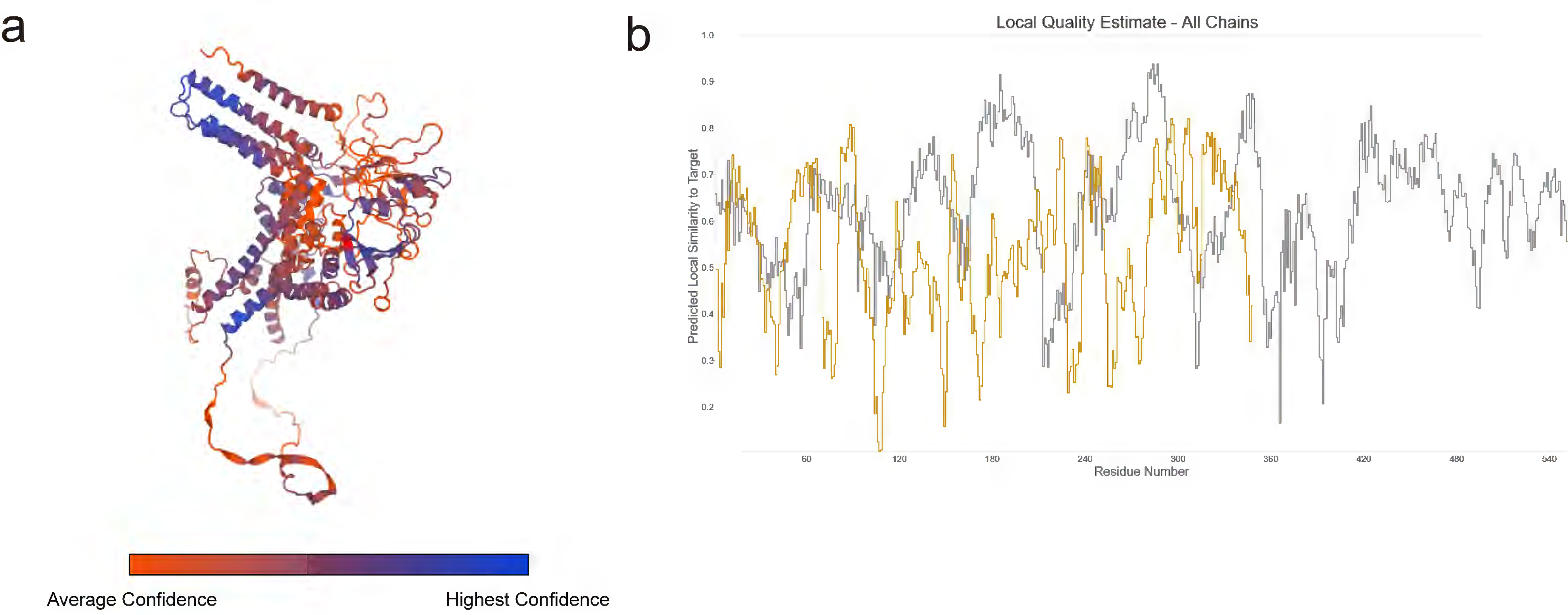

a
Average Confidence
Highest Confidence
b
Local Quality Estimate - All Chains
1.0
0.9
0.8
0.7
0.6
0.5
0.4
0.3
0.2
Predicted Local Similarity to Target
60
120
180
240
300
360
420
480
540
Residue Number

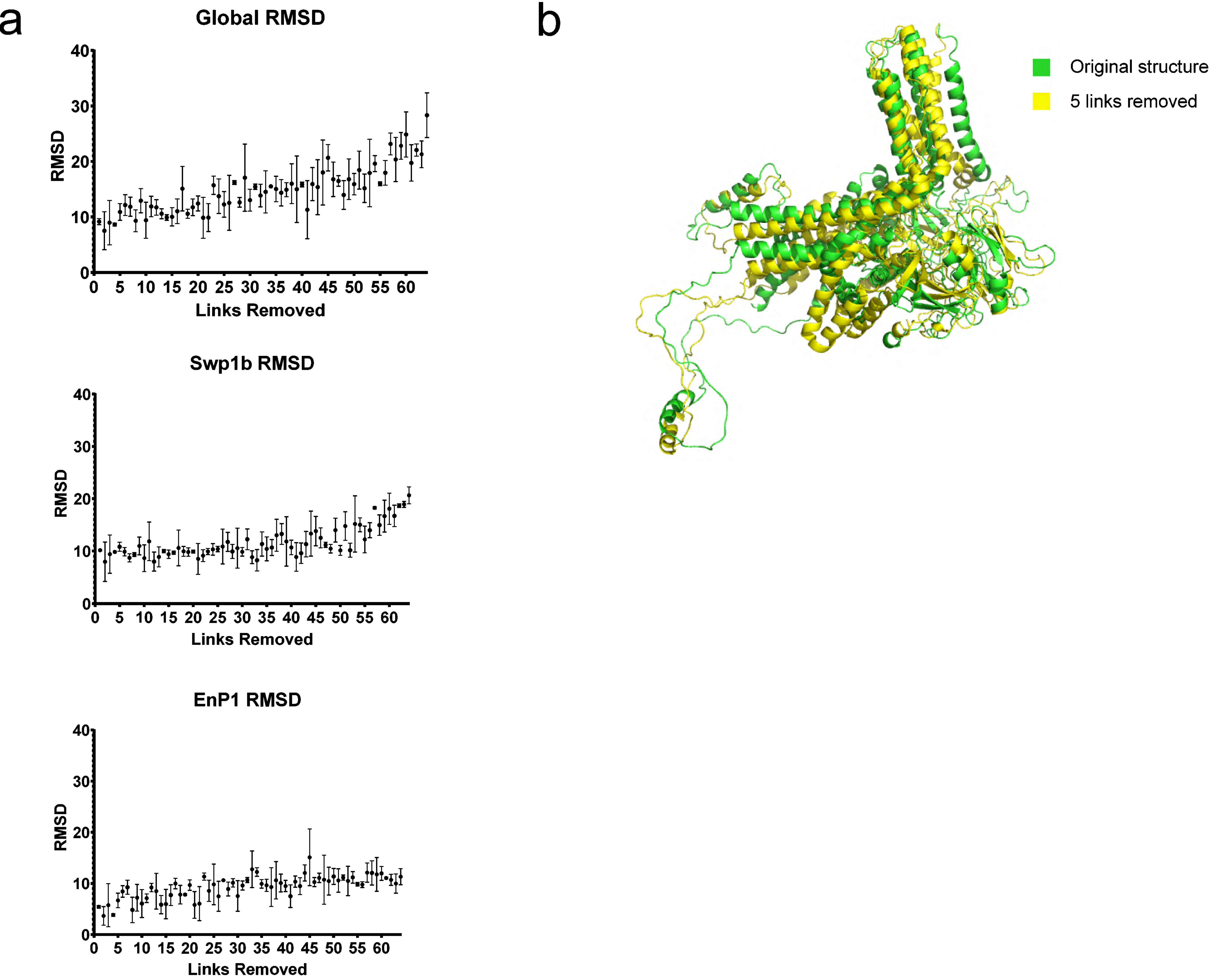

a
Global RMSD
RMSD
Links Removed
Swp1b RMSD
RMSD
Links Removed
EnP1 RMSD
RMSD
Links Removed
b
Original structure
5 links removed

Plasmids	Selective Media	Expected Result if Positive	Swp1b-FL EnP1-FL	Swp1b$^{32-353}$ EnP1$^{141-348}$	$(GGGGS)_3$-Swp1b-FL $(GGGGS)_3$-EnP1-FL	Swp1b$^{20-60}$ EnP1$^{141-215}$
**BD-52 AD-T (Positive Control)**	-Leu/-Trp	+	+	+	+	+
	-Leu/-Trp/XaG/Aba	+ blue	+ blue	+ blue	+ blue	+ blue
**BD-Lam AD-T (Negative Control)**	-Leu/-Trp	+	+	+	+	+
	-Leu/-Trp/XaG/Aba	-	-	-	-	-
**BD-Swp1b (test for autoactivation)**	-Trp	+	+	+	+	+
	-Trp/XaG	+ white or blue	+ blue	+ blue	+ blue	+ blue
	-Trp/XaG/Aba	-	-	-	-	-
**BD Empty (test for bait toxicity)**	-Trp	+	+	+	+	+
**BD Empty AD-EnP1 (test for false positive)**	-Leu/-Trp/XaG	+ white	+ blue	+ blue	+ white	+ white
	-Ade/-His/-Leu/-Trp/XaG/AbA	-	-	-	-	-
**BD-Swp1b AD-EnP1**	-Leu/-Trp/XaG	+ blue	+ blue	+ blue	+ blue	+ blue
	-Ade/-His/-Leu/-Trp/XaG/Aba	+ blue	-	-	-	-

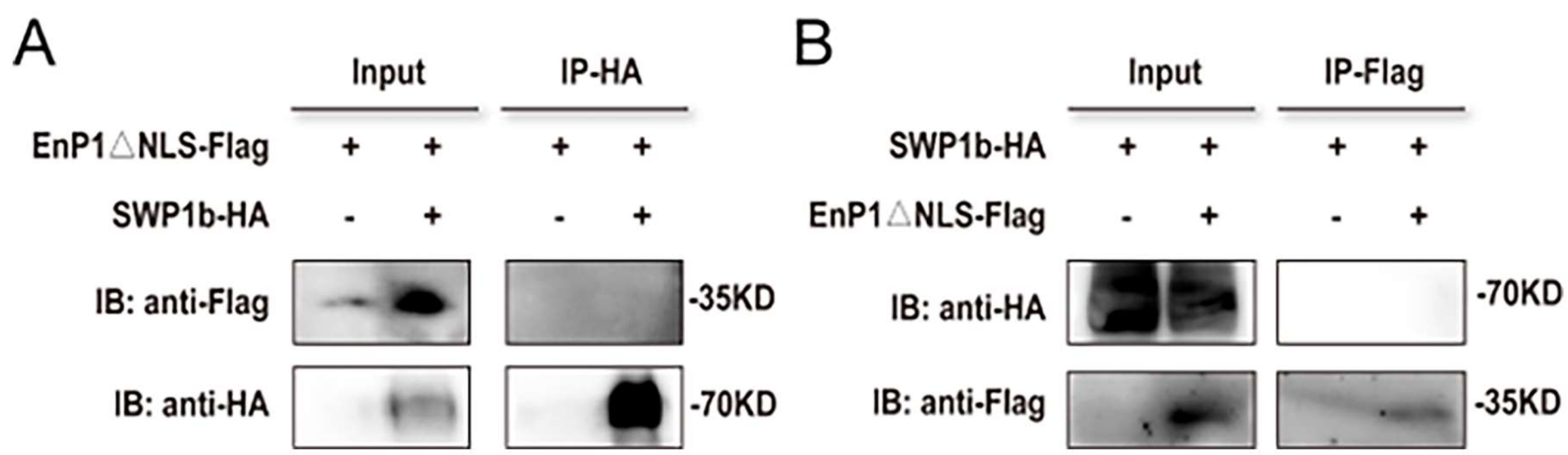
A
Input
IP-HA
EnP1△NLS-Flag
SWP1b-HA
IB: anti-Flag
IB: anti-HA
-35KD
-70KD
B
Input
IP-Flag
SWP1b-HA
EnP1△NLS-Flag
IB: anti-HA
IB: anti-Flag
-70KD
-35KD
