# Supplementary material for: Cross-linking mass spectrometry guided structure prediction of disordered Encephalitozoon hellem proteins
Source: mBio. 2026 Oct 20;17(11):e02261-26. doi: 10.1128/mbio.02261-26 (PMC13753748; doi:10.1128/mbio.02261-26)
Supplement: Supplementary file 2 [file mbio.02261-26-s0005.docx]

**SUPPLEMENTAL FIGURE LEGENDS**

**Supplemental Figure 1.** Number of crosslinks (intralinks and interlinks) found in each independent replicate that contributed to the Swp1b-EnP1 model. Created with Biorender.com.

**Supplemental Figure 2.** XL-MS crosslinks mapped onto Swp1b, EnP1, and Swp1b-EnP1 structures generated by ESMFold. Links below the 30Å cutoff are colored black, links above the 30Å are colored red. 128/224 crosslinks (57%) fall below the 30Å cutoff in the Swp1b-EnP1 complex.

**Supplemental Figure 3.** PAE plots from AlphaFold3 prediction of Swp1b, EnP1, and the Swp1b-EnP1 complex.

**Supplemental Figure 4. Analysis of Hydrogen bonds and Salt Bridges.** List of hydrogen bonds (H-bonds) and salt bridges between Swp1b and EnP1 atoms (left) and mapped onto the model (right).

**Supplemental Figure 5. Quality analysis of Swp1b-EnP1 model a.** Per-residue confidence as determined by QMEAN. **b.** Local quality estimate as determined by WHATCHECK.

**Supplemental Figure 6. Analysis of RMSD as links are removed.**

**a**. Global RMSD calculated by aligning entire structure. 3 random sets of links were removed, from removing 1 link to removing 64. Individual RMSD calculated by aligning individual chains.

**b**. Superposition of new model and model produced by removing 5 random interlinks.

**Supplemental Figure 7. Yeast-2-hybrid analysis of Swp1b and EnP1 interaction**. Four versions of Swp1b and EnP1 were tested. The full length of each protein (Swp1b-FL and EnP1-FL), approximately half of each protein (Swp1b^32-353^ and EnP1^141-348^), small pieces of the protein predicted to interact (Swp1b^20-60^ and EnP1^141-215^, and the full-length proteins with a flexible linker added to the N terminus ((GGGGS)_3_-Swp1b-FL and (GGGGS)_3_-EnP1-FL). All controls (first 10 rows) worked as expected. No interaction between Swp1b and EnP1 was detected in any of the four versions, as they failed to grow on -Ade/-His/-Leu/-Trp/XaG/Aba selective media.

**Supplemental Figure 8. Co-immunoprecipitation does not reveal an interaction between EnP1ΔNLS-Flag and SWP1b-HA** Co-immunoprecipitation (Co-IP) assays of the interaction between EnP1ΔNLS-Flag and SWP1b-HA. **a.** Lysates from cells co-expressing EnP1ΔNLS-Flag and SWP1b-HA were immunoprecipitated with anti-HA magnetic beads (IP-HA). Inputs and immunoprecipitates were immunoblotted (IB) with anti-Flag (upper panel) and anti-HA (lower panel) antibodies. **b.** Reciprocal co-IP assays using anti-Flag magnetic beads (IP-Flag). IB was performed with anti-HA (upper panel) and anti-Flag (lower panel) antibodies. Molecular weights (kDa) are indicated on the right. Experiments were independently repeated using HA magnetic beads and Flag magnetic beads, respectively.

**SUPPLEMENTAL DATA**

**Supplemental Data 1** List of *E. hellem* crosslinks

**Supplemental Data 2** List of Swp1b-EnP1 crosslinks

**Supplemental Data 3** List of independent set of crosslinks

**SUPPLEMENTAL, SCRIPTS, AND FILES**

**RRI_FDR.R**	Extract crosslink data from Annika2.0.

**getAlphaLinkFile.R** Extract intralinks for proteins of interest and put in AlphaLink format.

**modify_pdb.R**	Modify pdb file for MODELLER format.

**xl-for-modeller.R**	Export crosslinks in MODELLER format.

**xl-for-modeller-plus-intralinks.R** Export crosslinks and intralinks in MODELLER format.

**Swp1b-EnP1.pir** Example .pir file for bringing together two chains in MODELLER.

**model_w_restraints.py**	Example file to run MODELLER.

**calculateMultimerDistance.py**	Map and record crosslink distances in PyMOL.
